# Review on Excess Noise Measurements of Resistors

**DOI:** 10.3390/s23031107

**Published:** 2023-01-18

**Authors:** Daniela Walter, André Bülau, André Zimmermann

**Affiliations:** 1 Hahn-Schickard, Allmandring 9b, 70569 Stuttgart, Germany; 2 Institute for Micro Integration (IFM), University of Stuttgart, Allmandring 9b, 70569 Stuttgart, Germany

**Keywords:** measurements, 1/f noise, excess noise, noise index, resistor, resistor network, strain gauge, sensor, inkjet, nanoparticles

## Abstract

Increasing demands for precision electronics require individual components such as resistors to be specified, as they can be the limiting factor within a circuit. To specify quality and long-term stability of resistors, noise measurements are a common method. This review briefly explains the theoretical background, introduces the noise index and provides an insight on how this index can be compared to other existing parameters. It then focuses on the different methods to measure excess noise in resistors. The respective advantages and disadvantages are pointed out in order to simplify the decision of which setup is suitable for a particular application. Each method is analyzed based on the integration of the device under test, components used, shielding considerations and signal processing. Furthermore, our results on the excess noise of resistors and resistor networks are presented using two different setups, one for very low noise measurements down to 20 µHz and one for broadband up to 100 kHz. The obtained data from these measurements are then compared to published data. Finally, first measurements on commercial strain gauges and inkjet-printed strain gauges are presented that show an additional 1/f^α^ component compared to commercial resistors and resistor networks.

## 1. Introduction

When it comes to resistors, circuit designers have to choose out of a large variety of resistor technologies. Choosing the appropriate resistor depends on many parameters associated with it, such as resistance, tolerance, power rating, temperature coefficient and package, to mention only a few out of many. With increasing demands for precision electronics, such as those needed in sensor applications, high-resolution test equipment or high-stability references, the noise of resistors as an additional parameter plays an important role. While all resistors exhibit inevitable thermal noise, often referred to as Johnson noise, excess noise, also known as current noise, is highly dependent on the technology used to manufacture the resistor and the current flowing through it. To choose the best resistor technology for any given application, it is crucial to understand which technology contributes which amount of excess noise, a topic that is not solely of academic interest. Although a standard on how to measure excess noise in resistors was defined [1,2], improved resistor technology and decreased excess noise require improving test equipment for its characterization. While datasheet values given by manufacturers are often quite conservative or limited by the test equipment used, if specified at all, only a limited number of papers contain investigations on different resistors and resistor networks. Different setups have been used in different papers published to this date and addressing this topic. This makes it hard to decide which setup is suitable for which application. Furthermore, not every paper describes the used setup to a point that it is easy to reproduce.

Very low excess noise has been found for a few specific resistor technologies. Common sense states that carbon resistors exhibit the largest amount of noise, followed by thick film, then thin film and finally metal foil and wirewound resistors [3], but large differences can be observed within thin film resistors depending on the resistive material and substrate being used [4]. Excess noise has been investigated [5,6,7] and there is an understanding for its causes [8,9].

Motivated by new technologies to create resistors for sensor applications, such as inkjet- and aerosol-jet-printed strain gauges and temperature sensors, and how they compare to existing resistor technologies, the theoretical background on noise in resistors is summarized. A review of the methods used in different papers to characterize excess noise is given, results of excess noise that have been provided so far are compared, a method to characterize very low excess noise is presented and the results of our own measurements performed on resistors and resistor networks to reproduce already published results are shown. Furthermore, measurement results from a commercial strain gauge in thin film technology as well as an inkjet-printed strain gauge made from silver ink are presented.

## 2. Theoretical Background

The content of this paper starts with a summary of the necessary theoretical background to understand the topic of noise measurements in resistors. In the following, the wide field of noise is briefly introduced, especially the noise types important in resistors. Afterwards, resistor technologies and the noise associated with them are presented.

### 2.1. Noise

**Figure 1:**
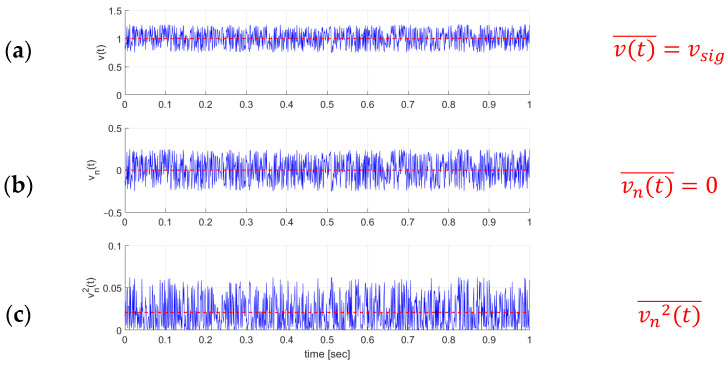
A common sensor signal can be divided into the actual sensor signal and the noise part. The sensor signal can be described with its mean value (**a**), while the mean value of noise will always be zero (**b**). Therefore, noise is characterized with its mean square value (**c**) (refer to [7]).

Every signal that is measured includes some kind of noise. According to [7], the voltage measured at a sensor output is

(1) $$v\left(t\right)=v_{sig}\left(t\right)+v_{n}\left(t\right),$$

where $v_{sig}$ is the actual voltage signal and $v_{n}$ is the voltage noise component. Figure 1a shows a measured signal and its arithmetic mean. The arithmetic mean is usually used to characterize the signal. The arithmetic mean of the noise is always $\bar{v_{n}\left(t\right)}=0$; see Figure 1b. Hence, it is more suitable to take the root mean square values for noise characterization (see Figure 1c) with the root mean square value given as

(2) $$v_{rms}=\sqrt{\bar{v_{n}{}^{2}\left(t\right)}}$$

which is usually used to quantify noise voltage [7].

Noise can be classified into extrinsic and intrinsic noise. Extrinsic noise describes all the noise affecting the system from the outside, e.g., from the environment. This might be natural noise sources such as sky noise as well as manmade noise or noise from power lines or electric motors [6]. This noise couples into the circuit either conductively or inductively. Intrinsic noise is the noise generated inside the system. The origin of this noise is the discrete nature of charge carriers [6]. The most relevant noise types for intrinsic noise are thermal noise, shot noise, burst noise, generation–recombination noise, excess noise (1/f-noise) and 1/f^2^-noise [9]. While thermal noise and excess noise are described in detail in the following due to their importance in the context of resistor noise, detailed information about other noise sources can be found elsewhere [6,7,8,9,10]. Noise types in general have two main characteristics. The first one is related to the physical phenomena, which are producing the noise. The second one is their frequency distribution [10]. Noise types are often associated with a color that corresponds to their distribution in the frequency spectrum.

#### 2.1.1. Thermal Noise

Thermal noise is the most prominent form of noise. It is also called Johnson or Johnson–Nyquist noise. Since its power spectral density is evenly spread over the whole frequency range, like the spectrum for white light, thermal noise is also referred to as white noise [9,11,12]. Another characteristic of thermal noise is its Gaussian amplitude distribution [12]. At temperatures above absolute zero, free electrons are moving in conducting materials due to kinetic energy. This inherent kinetic energy is proportional to temperature and therefore to thermal energy. Thereby, fluctuating charge levels occur at the ends of every resistor. This time-dependent noise voltage can be measured as thermal noise [6,7,13]. Thermal noise does not depend on material or the configuration of an electrical circuit but only on constants [14], and the voltage noise density of thermal noise that is constant over frequency [15] can be given by the formula

(3) $$e_{th}=\sqrt{4*k*T*R},$$

where $e_{th}$ is the effective voltage of thermal noise in a given bandwidth of 1 Hz, $k\approx 1.38*10^{-23}\frac{J}{K}$ is the Boltzmann constant, T is absolute temperature in K and R is resistance in Ω. Similar to the definition of voltage noise density, the short-circuit noise density *i_th_* is given by [15]

(4) $$i_{th}=\sqrt{\frac{4*k*T}{R}}$$

As Equation (3) indicates, there are only three possibilities to decrease voltage noise:

1. Decrease resistance,
2. Decrease bandwidth,
3. Decrease temperature.

**Figure 2:**
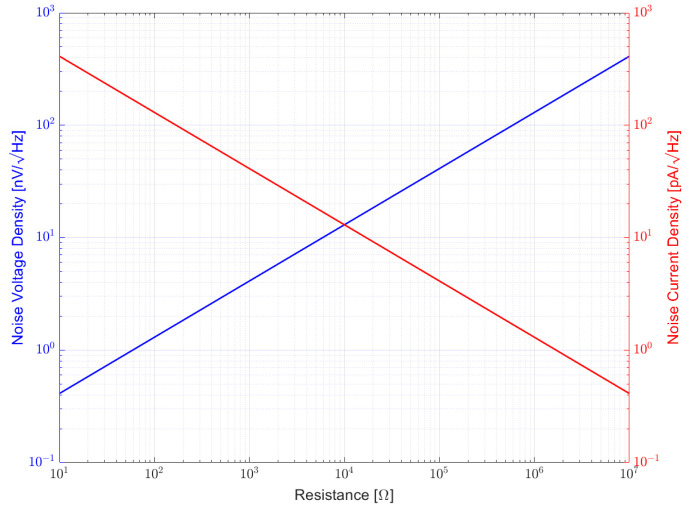
Voltage noise of a resistor increases proportional to its resistance with 10 dB per decade while noise current decreases with 10 dB per decade.

Figure 2 shows in a double logarithmic plot how noise voltage increases with resistance while current noise decreases with resistance. In fact, it is an increase and decrease, respectively, with 10 dB per decade.

#### 2.1.2. Excess Noise

Excess noise is also called 1/f-noise, flicker noise, current noise or pink noise. This type of noise cannot be described by using a single formula with some constants like thermal noise [1]. Hooge [5] shows in his review that covers the field of excess noise that many theories and models were proposed for excess noise. So far, no unifying principle can be identified that would explain this type of noise, though some sources have been determined [15]. By observing excess noise, some characteristics can be derived anyway. It is only present when current flows through the device [11,16]. As the name 1/f-noise already indicates, the power spectrum of 1/f-noise is inversely proportional to frequency and follows $\frac{1}{f^{\alpha }}$, where α≈1 [9,11]. Some publications even distinguish between fundamental 1/f-noise and 1/f^α^-noise with α=0.8−1.2 [17]. According to [18], in a noise density plot, excess noise $e_{f}$ can be described by

(5) $$e_{f}=\frac{K}{\sqrt{f}},$$

where K is a constant representing the noise density value $e_{f}$ at a frequency of f=1 Hz.

How excess noise behaves at very high and very low frequencies is of high interest but quite difficult to determine. At high frequencies, thermal noise always superimposes 1/f-noise, which makes it difficult to spot the further trend. The measurement of 1/f-noise at very low frequencies is very time-consuming, as f=0 Hz can never be reached [19]. Hooge [8] states that the spectrum cannot be exactly $\frac{1}{f}$ from f=0 to f=Infinity because of the mathematical rule that neither the integral of power density nor the Fourier transformation are able to have infinite values. This gives rise to the assumption that excess noise might not have a 1/f spectrum over the whole frequency range. However, in [15], Horowitz and Hill argue that excess noise increases forever. Excess noise in contrary to thermal noise has no Gaussian distribution in its power density function [6]. Additionally, excess noise is voltage-dependent, more precisely, it is proportional to the applied voltage [20] across a resistor. In general, excess noise is caused by a DC current flowing through a discontinuous medium, the interaction between charge carriers and surface energy, and imperfect contacts and crystal defects [6,7,9,17,21,22]. This means the magnitude of the excess noise spectrum is dependent on inherent properties of resistors such as material composition, processing technology, size and shape [1,17]. The level of excess noise is related to the quality of its lattice. Hence, high excess noise is an indication for poor material quality and low reliability [6]. To specify the level of excess noise inherent in a device, the noise index (NI) is used [21], which is explained in detail later.

#### 2.1.3. Noise in Resistors

**Figure 3:**
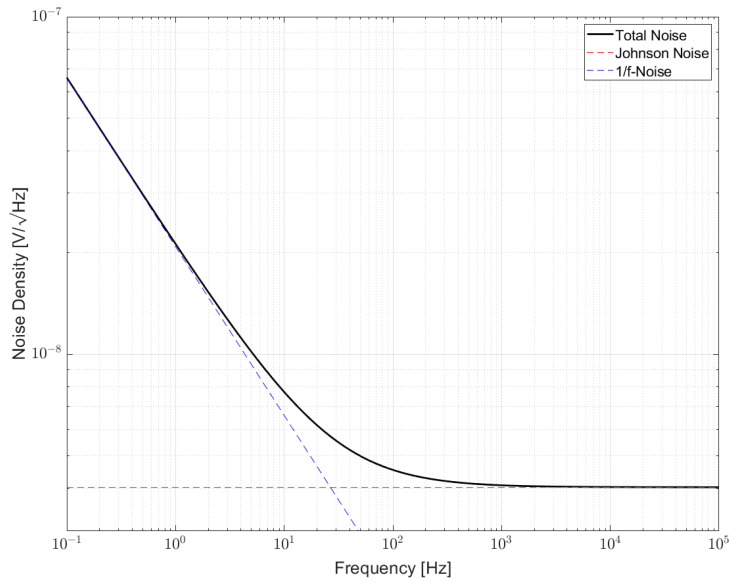
Noise in resistors is a combination of thermal noise and excess noise. At low frequencies excess noise dominates while at high frequencies thermal noise dominates.

This section introduces the combination of noise types occurring in a resistor. Figure 3 shows how the noise density of a resistor is distributed over frequency. The black curve shows the total noise in a resistor $e_{tot}$. It is a combination of thermal noise $e_{th}$ (red curve) and excess noise $e_{f}$ (blue curve). Since thermal noise and 1/f-noise are independent noise sources and therefore uncorrelated, noise densities cannot be added linearly. Instead, the sum of the squares has to be used to obtain total noise [10,11]

(6) $$e_{tot}{}^{2}=e_{th}{}^{2}+e_{f}{}^{2}.$$

As can be derived from Figure 3, excess noise dominates in the lower frequency range while thermal noise dominates at higher frequencies. The frequency where thermal noise has exactly the same value as excess noise is called corner frequency $f_{c}$. According to [15,18] it is also possible to describe the total noise by means of the corner frequency with

(7) $$e_{tot}=e_{th}*\sqrt{\frac{f_{c}}{f}+1}$$

Thermal noise $e_{th}$ can be calculated by using Equation (3). In this example, a resistance of 1 kΩ and an ambient temperature of 300 K were assumed. Using (4), 1/f-noise can be described. The constant K of a certain resistor can only be derived from measurements. The type of these measurements and their performance will be analyzed in chapter 3. As stated earlier, excess noise is dependent on material composition, technology, size and shape and, therefore, is an indication for the material quality of the measured resistor [1,6,17]. The slope of excess noise in the spectral density plot increases with 10 dB/decade or with a factor of 10 in two decades towards lower frequencies, since the energy is the same in any bandwidth. The area under the curve of the total noise density in Figure 3 over any given bandwidth $f_{1}$ to $f_{2}$ is the root mean square noise voltage. This can mathematically be described by the integral of the square of Equation (6) in the frequency band $f_{1}$ to $f_{2}$ and will result in [18]

(8) $$v_{n}=e_{th}*\sqrt{f_{c}*ln\frac{f_{2}}{f_{1}}+f_{2}-f_{1}}$$

The only way to decrease excess noise contribution for a given resistance is by reducing the current flowing through it.

### 2.2. Measurement Units to Express Excess Noise

As has been shown in the previous section, excess noise is represented by a linear slope in a double logarithmic noise density plot. From Section 2.1.2, it is already known that excess noise is dependent on the current through the resistor, which results from a supply voltage and the resistor being measured. Hence, it is necessary to find a measurement unit that describes the excess noise in a device under test independently from its supply voltage. For this purpose, different measurement units have been proposed. In earlier publications, the expression microvolts per DC voltage was mostly used to quantify excess noise. In particular, this is the ratio of the mean rectified resistor noise in µV to the DC voltage in volts applied to the resistor [23]. According to Conrad, Newman and Stansbury [23], this index was used in lack of better options. However, the suggested index is not comparable due to the often-missing specification of the pass band.

#### 2.2.1. Conversion Gain

In 1956, Conrad suggested a new index called conversion gain as a reproducible measurement unit that is independent of loading power, the used test equipment and test procedures [24]. Thereby, conversion gain $G_{C}$ describes the noisiness of a resistor and the efficiency of a resistor to convert applied DC voltage power $P_{dc}$ to current-noise power $P_{a}$. The power ratio of those named powers is given in dB [24]. The corresponding equation can be found in [23,24] as

(9) $$G_{C}=10*log\left(\frac{P_{a}}{P_{dc}}\right),$$

where $P_{a}$ is defined as the current-noise-power spectral density (NPSD) in microvolts-squared at 1 kHz of a resistor with a resistance of R and

(10) $$P_{a}=\frac{NPSD*10^{-12}}{4*R}$$

Thereby, the power of DC voltage applied to the resistor is defined as

(11) $$P_{dc}=\frac{V^{2}}{R},$$

where V is the applied DC voltage across the resistor. By combining Equations (9)–(11), the value of the resistance R does not have to be determined, which is convenient for measuring [24]. The drawback of the conversion gain is that there is no relation to the formerly used index µV-per-Volt and that values usually range from −140 dB to −200 dB, which is difficult for visualization [23].

#### 2.2.2. Noise Index

To overcome these drawbacks, the noise index (NI) was proposed and picked up by Conrad, Newman and Stansbury [23]. This index has a simple relation to conversion gain and is also familiar to µV-per-Volts. It is defined as the ratio of the rms noise voltage $v_{rms}$ (in µV) in a pass band of one frequency decade to the applied DC voltage V (in V) and is expressed in dB [23]. The corresponding formula is

(12) $$NI=20*log\left(\frac{v_{rms}}{V}\right).$$

In this expression, 1 µ*V_rms_* of noise in a decade together with a supply DC voltage of 1 V would correspond to a NI value of 0 dB. $v_{rms}$ has to be determined from measurements [11]. Since the DC voltage supply is part of the equation, it does not have to be selected carefully, and NI values can be compared anyhow [23]. It is possible to transfer noise index into conversion gain by

(13) $$NI-G_{C}=-159.6\;dB.$$

The exact derivation of this relation can be found elsewhere [23].

With Equation (12), NI can be calculated when $v_{rms}$ in a single decade is given or vice versa. Sometimes a certain value of noise density at a particular frequency might be of interest, e.g., to draw graphs. This eventually leads to Seifert’s work [22] where the mean-square noise voltage is given by

(14) $$\bar{v^{2}}=v_{rms}{}^{2}=\int_{f_{1}}^{f_{2}}S\left(f\right)df=\int_{f_{1}}^{f_{2}}\frac{\bar{e^{2}\left(f\right)}}{\Delta f}df$$

Thereby, S(f) is the power spectral density (PSD) of the resistor excess noise in the frequency band $f_{1}$ to $f_{2}$ and $\frac{\bar{e^{2}\left(f\right)}}{\Delta f}$ is the mean-square noise spectral density (NSD) at frequency f. According to Section 2.1.2, spectral density of excess noise is proportional to 1/f. Due to this reason, the product of the power spectral density and frequency can assumed to be constant and according to [22] Equation (14) can be rearranged to

(15) $$v_{rms}{}^{2}=\frac{\bar{e^{2}\left(f\right)}}{\Delta f}*f*\int_{f_{1}}^{f_{2}}\frac{1}{f}df$$

(16) $$v_{rms}{}^{2}=\frac{\bar{e^{2}\left(f\right)}}{\Delta f}*f*ln\frac{f_{2}}{f_{1}}$$

If one decade with the relation $f_{2}=10*f_{1}$ is considered as the frequency range, the part $\ln\frac{f_{2}}{f_{1}}$ equals ln10. Replacing this relation in Equation (16) and rearranging the equation in order to obtain the noise density at a certain frequency results in

(17) $$e_{n}\left(f\right)=\sqrt{\frac{\bar{e^{2}\left(f\right)}}{\Delta f}}=\frac{v_{rms}}{\sqrt{f*ln10}}$$

### 2.3. Resistor Types and Technologies

Since excess noise in resistors is dependent on resistor-specific characteristics, different resistor types and technologies are described in the following section.

#### 2.3.1. Resistor Types

Resistors come in a variety of different resistance values, sizes and other characteristics. In many applications, it is not the absolute resistance that is important, but resistor ratios, such as gain setting resistors in an amplifier [4,25]. To reach much better performance than with single resistors, resistor networks can be used. Resistor networks are multiple resistors in one package that are created with the same processes simultaneously, often on the same substrate. This leads to good resistance matching between the individual elements as well as small tolerances, good tracking of temperature coefficients and thermal coupling of elements within the network [4].

A special type of resistors for sensing applications are strain gauges. In contrary to the already mentioned fixed resistor types, strain gauges are variable resistors [25]. Due to experienced strain, they alter their resistance. Strain gauges can be used as a single element or in a bridge configuration, e.g., in a Wheatstone bridge.

#### 2.3.2. Resistor Technologies

As mentioned in Section 2.1.2, excess noise is dependent on resistor technology. It is common sense that carbon composition resistors show the largest excess noise, followed by thick film and thin film resistors. Metal foil and wirewound resistors exhibit the smallest excess noise.

In carbon composition resistors, the whole body acts as the resistive element [26]. They are produced by mixing carbon particles and a special binder and compressing them to a solid element. At their ends, termination wires are attached. The whole resistor is sintered in a furnace [6,26]. Since no trimming is carried out, composition resistors have large tolerances in the range of ±10% and ±20%. Their advantages are a good high-frequency characteristic as well as the capability to be overloaded relative to their size. Composition resistors are often used in power supplies, welding controls or as “dummy loads” [26].

Thick film resistors are mostly produced as SMD devices [25]. They are fabricated by screen-printing resistive pastes on a ceramic substrate. The resistive paste contains powders (e.g., silver, chromium, palladium, glass). The printed paste is then sintered in a furnace at about 800 °C in order to evaporate the organic components and melt the glass particles to form a stable resistor layer [6,27]. With automated processes, thick film resistors can be produced in large quantities and their reproducibility is good [25]. Due to the junctions between metallic grains and glass particles, intrinsic defects in the conducting layer are present and thick film resistors exhibit large excess noise [17].

Thin film resistors come with many different materials for the resistive layer, e.g., nickel–chromium, carbon–boron, tantalum, tantalum–nitride, various oxides and other alloys are used [6,25]. The thin film layer can be deposited on the substrate using different technologies such as ion deposition, sputter deposition, chemical vapor deposition and evaporation [25]. The film thickness is around 0.5 µm and the film is usually patterned and laser-trimmed to increase and adjust the resistance value [6,25]. The substrate can be an alumina-based ceramic, sapphire or surface-oxidized silicon [25]. Within the class of thin film resistors, the electrical properties including excess noise can be different depending on the thin film and substrate materials used [4,26]. Metal film resistors as a category of thin film resistors are supposed to have the best noise properties but are still worse than bulk metal foil or wirewound technology due to occlusions, surface imperfections and non-uniform deposition [3].

Metal foil resistors are made of a pure metal or metal alloy foil on a carrier that is attached to a solid ceramic or glass substrate. As resistive material, nickel–chromium is often used. The resistive layer is patterned with a meander by using photolithography and etching [6,25]. To obtain the desired resistance value, connections in the pattern are cut with a laser beam. Each part of the pattern and its respective resistance is well known in order to trim the resistor according to the desired value. An algorithm shows the connections to be cut for a specific resistor value [25]. Metal foil resistors are available from the mΩ to kΩ range [26]. A great advantage of metal foil resistors is their outstanding temperature coefficient. There are two important effects. First, the resistance of the foil increases with increasing temperature. Second, the coefficient of thermal expansion of the substrate is smaller than the coefficient of thermal expansion of the foil. With increasing temperature, this leads to compressive stress of the foil and decreases resistance. Due to these opposite effects, the resulting change in resistance is almost zero when temperature changes [25]. Metal foil resistors are used for low-ohm currents called shunts and precision resistors for measurement applications [26].

Wirewound resistors consist of a wire made up of a metal alloy that is wound onto a bobbin that might be made up of plastic, glass or ceramic. The wire ends are soldered, crimped or welded to the leads and the whole body is coated with a protective glaze [6,25], coated with silicone and either molded or inserted into a plastic shell and potted. For applications with high demands, hermetic packages with glass feedthroughs are used. Due to their processing technology, they can be very stable also at high temperatures over 200 °C [25,26]. A drawback of wirewound resistors is their inductive behavior that also makes them a bad choice for high frequency applications [26]. Reactances do not generate noise in general, but if current noise is running through them, they develop voltage noise and associated parasitics [3]. To obtain non-inductive wirewound resistors, two identical wirings can be used that run in opposite direction [25]. Noise characteristics of wirewound resistors are comparable to that of metal foil, but wirewound resistors show much more inductive behavior [3].

## 3. Measurement Techniques

**Figure 4:**
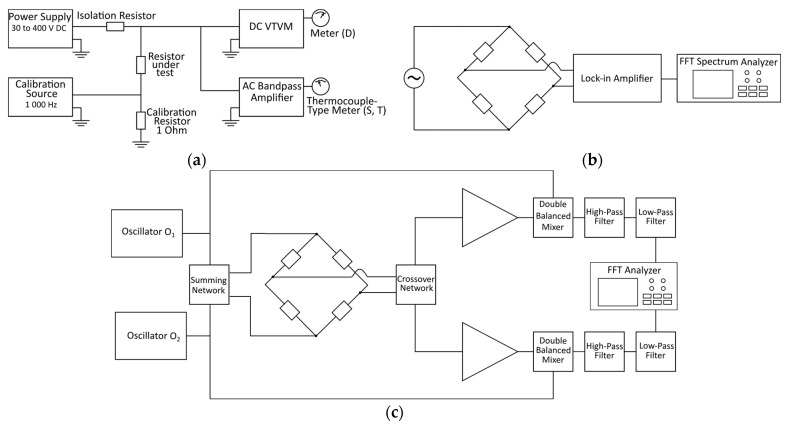

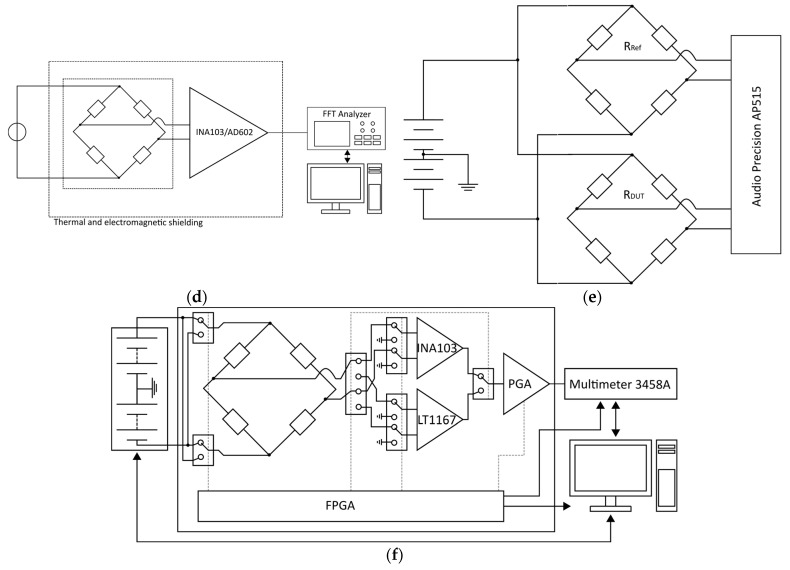
Block diagrams of different setups from the literature: (**a**) proposed standard method in [23], (**b**) setup in [35], (**c**) setup in [33], (**d**) setup in [22], (**e**) setup in [11] and (**f**) setup in [4].

In the following section, different measurement techniques from the literature are presented. The standard method is presented first, and it is discussed why it is not recommended to measure modern components. The other methods are analyzed according to the following parameters: usage of a bridge setting, voltage supply, low-noise amplifier, shielding and measurement device. Block diagrams of selected papers are depicted in Figure 4. A summary of the parameters is provided in Table 1.

### 3.1. Standard Method

The standard method is based on the paper of G. Conrad, Jr., N. Newman and A. Stansbury with the title “A Recommended Standard Resistor-Noise Test System” from 1960 [23]. The introduced method was transferred to the currently known standard methods MIL-STD-202-308 [1] and IEC60195 [2]. Method 308 describes a resistor test method to establish the noise quality characteristics and helps to choose a suitable resistor when current–noise requirements exist [1]. The setup consists of several parts that are depicted in the block diagram of Figure 4a. The first part is a variable DC power supply. The resistor under test is supplied through an isolation resistor. The latter one prevents noise appearing at the terminals of the resistor under test from being attenuated by the very low parallel impedance of the output terminals of the DC power supply. The isolation resistor is ideally free of current noise. Low noise wirewound resistors with values of 1 kΩ, 10 kΩ, 100 kΩ or 1 MΩ depending on the value of the resistor under test are used. Standard nominal values for DC voltage and voltage for the isolation resistor are given in a table in [1]. The setup also consists of a DC vacuum-tube voltmeter (VTVM). It measures the DC voltage across the resistor under test and its resulting noise (D). The voltage noise at the resistor under test is amplified and is shown by the AC indicating amplifier. The amplifier characteristics are high gain and low noise. The filter included has a 1 kHz pass band and is centered at 1 kHz. The setup is recommended to be shielded and placed at an ambient temperature of 25 ± 2 °C. The whole measurement consists of three steps. First, a calibration is needed. Then, the “open circuit” current–noise voltage of the resistor under test is measured. Afterwards, the system noise (S) is measured, followed by a simultaneous measurement of the DC voltage and the resulting total noise (T). By subtracting all components from the total noise, the noise of the resistor under test can be determined. The noise of the resistor under test can be expressed in the ‘microvolts-per-volt-in-a-decade’ index

(18) $$index_{dB}=T-f\left(T-S\right)-D$$

(19) $$\mathrm{with}\;f\left(T-S\right)=-10*log\left(1-10^{-\frac{T-S}{10}}\right).$$

This noise quality index is expressed in dB. The essential drawback of this method is the use of an analog filter with a specified passband. The noise measured in this frequency band contains all sources of noise. Modern low excess noise resistors might be dominated especially by thermal noise in this pass band. This method cannot give an answer as to which noise is actually measured [11]. Additionally, a low-noise isolation resistor is used. However, it still contributes to the total noise, most notably if the resistor under test is also low-noise. Nevertheless, this method is still widely used by resistor manufacturers, although its sensitivity is poor, especially for modern low-power precision components [4]. This is also the reason why manufacturers only give an upper limit between −30 and −40 dB for excess noise in their datasheets. One exception is the LT5400, which is specified with a noise index of <−55 dB [4].

As shown in the current section, the standard method has some disadvantages and requirements on the noise of resistors have increased over time. Excess noise has greater and greater meaning for measurements. Today’s high-quality resistors cannot be measured with this test method due to several limitations. For this reason, other methods can be found in literature, such as bridge configurations. Important parameters that will be discussed in the following are the bridge setting of resistors, voltage supply, used amplifiers, shielding of measurement setup and measurement equipment. Table 1 gives a summary of the most important papers on noise measurements. Other papers were found, but they had little information given on the measurement setup [17,28,29]. The papers are sorted by year of publication starting with the work of G. Conrad, Jr., N. Newman and A. Stansbury [23], which is the basis for the current standard method. There are other papers before 1960 where similar methods were used [30,31].

### 3.2. Bridge Setting

As shown in the previous section, the standard method uses a single resistor as the resistor under test in series with a wirewound resistor. This will work properly under the assumption that the wirewound resistor is noise-free or at least of lower noise than the resistor under test. To overcome this issue most papers since 1980 used a Wheatstone bridge configuration for the resistor under test, as indicated by the column ”bridge setting” in Table 1. Some of the papers used a bridge configuration with two sample resistors and two ballast resistors [10,32], but the prevalent method was to use four “identical” sample resistors [4,11,20,22,33,34,35,36,37]. The bridge as a differential configuration has the advantage to be able to suppress disturbances caused by the power supply and other common mode interferences [22,35]. In general, the bridge consists of four resistors. Bridge bias voltage supply is applied to one diagonal of the bridge; therefore, two dividers are fed by the same voltage [25]. Every half bridge experiences the full bias voltage of the Wheatstone bridge. Hence, the voltage across one resistor is half of the bridge bias voltage [22]. This is important when calculating the NI in Equation (12), where DC voltage V corresponds to the voltage drop across one resistor of the bridge. The voltage fed into the amplifier is picked up over the second diagonal of the bridge. If the bridge is totally balanced, this voltage would be exactly zero. In practice, the voltage measured across the second diagonal of the bridge for resistors with the same nominal value and a voltage noise of *v_rms_*_,1_, *v_rms_*_,2_, *v_rms_*_,3_ and *v_rms_*_,4_ is according to [37]

(20) $$\bar{\Delta v_{rms,tot}}=\sqrt{\frac{v_{rms,1}{}^{2}+r_{rms,2}{}^{2}+v_{rms,3}{}^{2}+v_{rms,4}{}^{2}}{4}}$$

Since this is the resulting voltage noise of all four resistors in the bridge, the factor $\sqrt{4}=2$ needs to be considered for voltage noise *v_rms_* of one resistor when calculating the NI value in Equation (12). Otherwise, as stated by Seifert in [22], ∆*v_rms,tot_* can be interpreted as the voltage noise of one resistor driven by the whole bias voltage of the bridge.

In Figure 4, some block diagrams of setups published in the literature using a Wheatstone bridge configuration are depicted. In Figure 4b, the setup used by Scofield [35] is shown, using AC excitation for thermal electromagnetic force (t.e.m.f.) cancellation, a commercial lock-in amplifier and a commercial FFT spectrum analyzer to measure and analyze the bridge voltage. The cross-correlation method with AC excitation is used by Stoll in [33] and displayed in Figure 4c. Besides a commercial FFT analyzer, it uses a fairly complex custom circuit. Seifert [22] uses a fairly simple setup with DC excitation and the bridge voltage measured using an instrumentation amplifier connected to an FFT analyzer as shown in Figure 4d. LaMacchia and Swanson [11] use a bipolar battery supply and a commercial high-performance audio analyzer for data acquisition, shown in Figure 4e. Finally, Beev [4] uses a bipolar polarity reversible DC excitation of the bridge, combined with cross-correlation using an 8.5 digit multimeter as a fast sampler, shown in Figure 4f. A field-programmable gate array (FPGA) is used to synchronize and control the switches of the bride supply, the amplifier input, the programmable amplifier and the multimeter.

**Table 1:** Summary of different measuring methods and their characteristics.

Paper	Year	Bridge Setting	Power Supply	Amplifier	Measurement Range	Analyzed Resistors	Measurement Device	Shielding	Techniques to Improve Measurement
Conrad et al. [23]	1960	✕	DC power (DUT) + ac power (ampl.)	AC band-pass amplifier (1 kHz)	At 1 kHz	100 Ω–22 MΩ	VTVM	Shielded enclosure for DUT	-
Hawkins and Bloodworth [38]	1971	✕	-	AC coupled amplifier	10 Hz–5 kHz	Thick film resistor	Homodyne spectrum analyzer	-	-
				Digital technique	1 × 10^−4^ Hz–4 Hz		-		
Stoll [33]	1980	✓	AC current	Ampl. (not specified)	-	-	2-channel Fourier analyzer	-	Cross-correlation method
Demolder et al. [34]	1980	✓	DC current, batteries	PAR 113	0.1 Hz–1 kHz	1 Ω–1 kΩ	-	Twisted cables, metal shield around setup	-
									Cross-correlation method
Scofield [35]	1987	✓	AC current	PAR 124A lock-in amplifier	0.1 Hz–100 Hz	Thin continuous metal films	Spectrum analyzer	Bridge in aluminum box surrounded by Styrofoam	-
Verbruggen et al. [36]	1988	✓	AC current with 45° phase shift	PAR 113	0.3 Hz–60 Hz	Thin film Al-sample	2-channel Fourier analyzer	-	-
Moon et al. [32]	1992	✓	AC current	Stanford SR560/PAR 116	0.1 Hz–100 Hz	480 Ω	Digital signal processor + PC	-	Digital mixing of supply current with 0° or 90° (or ±45°)
Leon and Hebard [10]	1999	✓	AC voltage	Lock-in amplifier	0.1 Hz–1 Hz	1 kΩ carbon composite	Spectrum analyzer	-	-
Crupi et al. [39]	2006	✕	-	OP27	1 Hz–1 kHz	1 kΩ	Spectrum analyzer	-	Cross-correlation method, different amplifier configuration measurements
Seifert [22]	2009	✓	DC voltage, battery	INA103	1 Hz–30 kHz	100 Ω, 1 kΩ	FFT analyzer	Aluminum box	-
				AD620		10 kΩ			
Maerki [20]	2013	✓	DC voltage, battery	AD8676 + INA103	0.1 Hz–1 MHz	470 Ω, 1 kΩ, 10 kΩ, 50 kΩ, 100 kΩ, 200 kΩ	-	-	Trimmer to zero bridge offset
				INA103					
Maerki [40]	2016	Half bridge	DC voltage	Differential amplifier 2015 dc	-	1 MΩ–50 GΩ	-	-	-
LaMacchia and Swanson [11]	2018	✓	Bipolar DC voltage, battery	-	5 Hz–40 kHz	2 kΩ	AP515	Cookie tin	Amplifier can be added for parts with low noise
Miyaoka and Kurosawa [37]	2019	✓	Bipolar DC voltage, battery	AD620 + AD797	1 Hz–100 kHz	10 kΩ (different technologies)	Oscilloscope	-	-
Beev [4]	2022	✓	Bipolar battery-based DC voltage	INA163 + PGA	0.001 Hz–10 Hz	Resistor net-works < 10 kΩ	HP 3458A digital voltmeter	Die-cast aluminum box, steel shielding box for carrier board and instrumentation amplifier	Correlated double sampling method with opposite bridge bias polarities, temperature stabilization
				LT1167 + PGA		Resistor net-works ≥ 10 kΩ			

### 3.3. Voltage Supply

The resistor under test for a single resistor as well as in a bridge configuration needs to be driven by a voltage to generate excess noise. Different possibilities are used in the papers mentioned in Table 1. It is conspicuous that most papers before the year 2000 used an AC voltage supply [10,32,33,35,36] in their setup while after 2000 all papers used a DC voltage supply [4,11,20,22,37,40]. The advantage of using AC supply is that resistance fluctuations are shifted to frequencies near the carrier frequency of the AC signal and therefore away from DC, where the excess noise of the amplifier is much greater than at higher frequencies [32,33]. Using two oscillating signals with a phase shift of 0° and 90° (or ± 45°) and performing measurements in a two-channel device helps to further decrease background noise [36]. Alternating supply voltages, on the other hand, have the advantage of eliminating contributions of thermal electromagnetic forces. The advantage of AC voltage supply at the same time is the disadvantage of DC voltage supply as amplifiers are noisier with DC [10]. Nevertheless, DC technique is predominant in newer papers since amplifiers improved over the years and exhibit low noise today [22].

The usage of batteries for DC voltage supply is prevalent [4,11,20,34,37] since they add the least additional disturbances to the signal. Some papers use an unipolar supply voltage [20,22] while others use a bipolar supply voltage [4,11,37] with a symmetrical arrangement to keep the bridge output near zero and further decrease common mode signals [4]. As investigated by Seifert [22], excess noise is dependent on the supply voltage. When specifying the NI value with Equation (12), supply voltage is already considered and NI values can be compared between setups of different supply voltage. It gets more challenging when comparing diagrams with measured noise densities since they have different absolute values when supplied with different voltages. Furthermore, mostly no limits for specified NI values are depicted in the diagrams, which makes it even harder to compare. To give a demonstrative example: Beev [4] used 10 V per element while Seifert [22] used 5 V per element. If the NI is calculated, they can be directly compared. The diagrams in the case of excess noise are different by a factor of two. This needs to be considered when comparing the results.

### 3.4. Low-Noise Amplifier

Measuring noise is associated with the measurement of small signals. This makes the usage of an amplifier mandatory for most setups. A subsequent requirement to the amplifier in a noise measurement setup is that the amplifier contributes as little noise as possible. Table 1 specifies the amplifier used in the respective papers. One exception is [11], where no amplifier is used. However, it is stated that for more sensitive measurements, an amplifier is a necessary improvement for the setup. While in the 1980s and 1990s, low-noise preamplifiers [32,34,36] and lock-in amplifiers [10,35] were used, more recent papers use low-noise operational [39] or instrumentation amplifiers [4,20,22,37] that come as integrated circuits. Instrumentation amplifiers have differential inputs and a single-ended output. Classically, an instrumentation amplifier consists of three operational amplifiers. They have several desirable characteristics such as very high input impedance (10 MΩ–10 GΩ) and a wide gain range (G = 1–1000). Additionally, they have a very high common mode rejection ratio (CMRR), especially at higher gains [15,19]. A disadvantage in using instrumentation amplifiers is the maximum allowed offset voltage at its input, resulting in a maximum allowed tolerance of the resistors in the bridge to not drive the output of the amplifier into saturation [22].

Operational amplifiers suffer from voltage and current noise, hence, for a given bridge resistance the adequate amplifier has to be chosen to account for both noise sources [21]. As can be seen in [4,22], different instrumentation amplifiers were used for different ranges of resistance values. This is because at low resistance values, voltage noise has the greatest impact on the overall noise of the amplifier, while at high resistance values, the current noise of the amplifier dominates the overall noise [19]. Therefore, for low resistance values, an instrumentation amplifier with low voltage noise might be chosen and for high resistance values, an instrumentation amplifier with low current noise might be required.

### 3.5. Shielding

The standard method only mentions that adequate shielding is necessary [1]. Shielding is inevitable since excess noise measurements are challenging. The aim of shielding is to keep away unwanted electromagnetic radiation from the outside. Otherwise, unwanted signals can appear in the measured signal. Machinery, power lines or computers are known sources for such radiations [13]. The purpose of an electrical shield is to protect the important parts from disturbances and to couple into ground [19]. A simple remedy can be found by isolating the measurement setup with a Faraday cage and using batteries for the power supply [13]. Grounded shields around wires or twisted wires can help to reduce capacitive or inductive coupling, but grounding of different parts has to be performed carefully in order to not create ground loops [19]. As shown in Table 1, there are measurement setups that use batteries such as in [4,11,20,34,37] for exactly this purpose. The column shielding reflects what the respective papers did to protect their measurement setup from unwanted signals. There can be found twisted cables [34] as well as aluminum boxes. The cheapest way is to use a tin can to create a Faraday cage [4,11,22,34,35].

### 3.6. Measurement Equipment

There are different possibilities for recording the data. Some papers use a voltmeter [1,4] or an oscilloscope [37], but most papers use fast Fourier transformation (FFT), digital signals or spectrum analyzers [10,22,33,35,36,38,39]. In some rare cases, modern audio cards are used [11]. For voltmeters and similar acquisition devices, the FFT has to be performed subsequently on a computer. One drawback of the standard method is that only the noise at 1 kHz is measured. By finding the FFT of a signal, information on noise over a wide range of frequencies can be obtained. This provides more insight into the behavior of noise [11]. In any case, the noise floor of the measurement device has to be taken into consideration.

To improve measurement resolution, two techniques were identified from the papers introduced in Table 1. The first method uses a correlation method. With the cross-correlation technique used in [32,33,34,39], uncorrelated noise as noise from the amplifier can be suppressed. Beev [4] introduced the correlated double sampling (CDS) method for resistor noise measurements, which suppresses parasitic components near DC, amplifier drift and excess noise of the amplifier itself. Additionally, Beev [4] used a PT1000 sensor and a thermoelectric cooler to thermally stabilize the measured resistors in order to suppress fluctuations due to self-heating of the sample or temperature variations of the environment.

Depending on the measurement setup, the measured noise density spectrum is not necessarily the noise density of the measured resistor. This is only true if no further noise is added by the measurement setup, e.g., the noise of the amplifier is orders of magnitudes lower. In case the noise of the amplifier is in the order of the device under test, voltage noise density and current noise density of the amplifier have to be determined and subtracted from the measured signal. In the case of uncorrelated noise sources, their powers are added [3,6].

## 4. Measurement Results

### 4.1. Most Important Measurement Results from Previous Publications

Many previous papers published measured data of one or more resistors. Table 1 gives an overview of measured resistance values and resistor types in the respective papers. Often, only the technology of the analyzed resistors and their resistance value are given without the exact part number, like is done in [37]. These results give a raw impression in which range the noise of a certain technology can be expected, but do not reflect the noise of a specific resistor series. Furthermore, in most cases, only the graphical results are given, not the noise indices. This makes it hard to compare the results to other measurements and even more if the supply voltage is not specified or the setup is completely different. The papers of Seifert [22] and Beev [4] solely show comprehensive results on specific resistor series. Table 2 summarizes a few simplified results from both papers in terms of a range for the NI, similar to how Beev presented his results of resistor networks. Additionally, he distinguished between different resistive materials and different substrates. In contrary, Seifert only presented diagrams in his paper with resistor values of 100 Ω, 1 kΩ and 10 kΩ. Some of their results were selected to be presented in Table 2.

### 4.2. Our Measurement Results

Motivated by new technologies to create resistors for sensor applications, such as inkjet- and aerosol-jet-printed strain gauges and temperature sensors, and the question of how they compare to existing resistor technologies, we performed our own measurements on commercial resistors. Therefore, noise measurements of commercial resistors given in the papers mentioned above were reproduced to verify the measurement equipment. Two different setups were used, the first one based on the setup of the Seifert paper [3], while the second setup used a nanovoltmeter. In the following, the analyzed resistors and their characteristics are given, the different setups are described and the results presented.

#### 4.2.1. Analyzed Resistors

The measurements were performed on different resistor types. The first category was single resistors with values of 100 Ω, 350 Ω, 1 kΩ and 10 kΩ. Table 3 lists all the analyzed single resistors with parameters such as manufacturer part number, resistance value, tolerance, power and resistor technology, if specified by their datasheets. In case the resistors of the same series with different resistance values were available, they are grouped in the table. All these resistors were measured with the first setup except for the 10 kΩ metal film resistor, which was analyzed with the second setup. The measurement setups are introduced in the following section.

Table 4 lists the analyzed resistor networks. In addition to the parameters from Table 3, there is an additional column with the material used for the resistor network since most datasheets contain this information. Again, resistor networks of the same series with different resistance values are grouped in the table. The resistor network with 100 Ω was measured with setup 1, the 1 kΩ networks with both setups and the 10 kΩ networks with setup 2 only.

Furthermore, strain gauges were analyzed in this paper. Table 5 lists the three strain gauges and their parameters. The first strain gauges were commercial 1-LY11-6/350 from Hottinger Brüel & Kjaer GmbH (HBK) with a nominal resistance value of 350 Ω and were free of strain. Additionally, strain gauges of type 1-XY33-6/350 that were attached to an aluminum substrate were measured. The third strain gauges were inkjet-printed with silver ink (Sicrys^TM^ I30EG-1) on a polyimide substrate and were sintered at 200 °C for 120 min. For the bridge configuration, the strain gauges with the four best matching resistances were selected. The average resistance value of the four selected strain gauges was 468 Ω. The strain gauges were measured with both measurement setups.

#### 4.2.2. First Setup: Measurement of Resistor Noise between 0.1 Hz and 100 kHz

**Figure 5:**
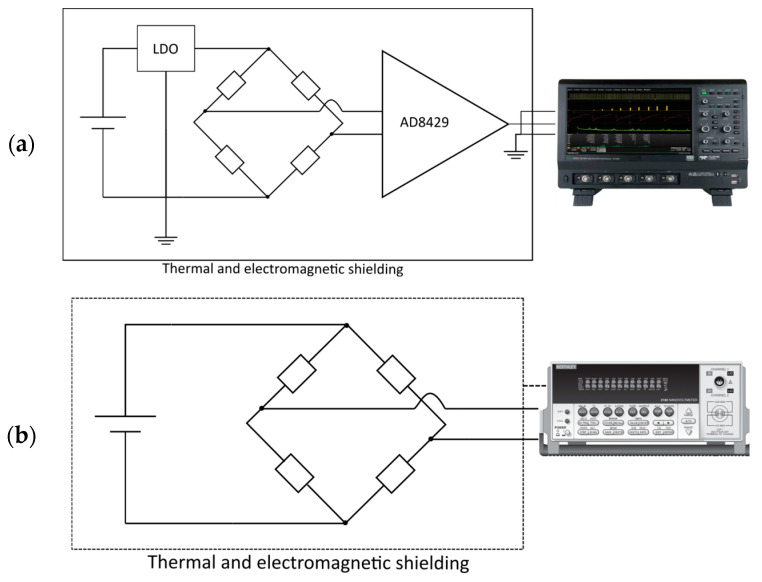
Block diagram of measurement setups used for measurement of noise in resistors. (**a**) corresponds to setup 1 with an AD8429 as instrumentation amplifier and an oscilloscope to record measurement values. (**b**) corresponds to setup 2 with a nanovoltmeter for capturing data.

**Figure 6:**
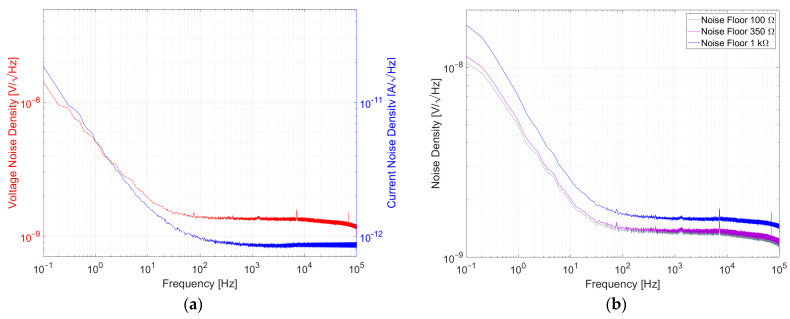
(**a**) Voltage noise and current noise of AD8429 and (**b**) total noise from the amplifier for different resistor values.

For the first setup, the resistors under test were arranged in a Wheatstone bridge. The setup was a variant of the setup used in [22], but with a different instrumentation amplifier and an oscilloscope instead of an FFT analyzer. To easily swap the resistors under test, they were mounted on pin headers, as shown in [7]. The bridge was powered by a 10 V supply voltage at the first diagonal. This means that every resistor in the bridge was biased with 5 V. The voltage was battery-based with two 6 V-cells in series, regulated to 10 V with a low-drop-out regulator (LDO) type LT3045. The resulting voltage across the second diagonal of the bridge was connected to the inputs of an AD8429 low-noise amplifier (LNA). The amplifier itself was battery-powered with ±12 V by two 6 V batteries in series for each rail. The gain was set to 60 dB. The whole setup was placed inside a tin can for electromagnetic shielding. The amplified signal was forwarded to a 12 bit Teledyne LeCroy Oscilloscope HDO6054-MS. Figure 5a shows a block diagram of the described setup. The signal was measured over a time period of 10 s. The oscilloscope has the ability to perform fast Fourier transformation (FFT) and rescaling to calculate the power spectral density as shown in [8]. Afterwards, the square root was calculated to obtain the noise density in V/√(Hz). An average of 100 measurements was taken. Hence, the measured result is a combination of resistor noise, amplifier voltage noise and amplifier current noise. To determine the noise contribution of the amplifier, the inputs of the amplifier were shorted for voltage noise measurement. The current noise of the amplifier was determined with a LT5400 100 kΩ resistor network, which is known for having very low excess noise. The measured voltage and current noise of AD8429 are depicted in Figure 6a. Figure 6b shows the contribution of voltage and current noise of the amplifier for different resistor values.

This contribution had to be subtracted from the measurement results. This subtraction was performed with GNU Octave on a computer after each measurement. The setup could measure noise of resistors with values between 100 Ω and 1 kΩ. The frequency span used for the investigation was 0.1 Hz to 100 kHz.

#### 4.2.3. Second Setup: Measurement of Very-Low-Frequency Resistor Noise

For very-low-noise resistors and measurements down to the mHz and µHz regime, a different setup was used. For 10 kΩ resistors, the bridge was fed by a battery-powered 10 V, low noise voltage reference based on a temperature-compensated, oven-controlled LTZ1000CH Zener that could provide up to 10 mA. For resistors ≤1 kΩ, the bridge was fed by 10 V from a low-noise linear dropout regulator type LT3045, which itself was powered by a 12 V battery (Figure 5b). The bridge voltage was measured using a Keithley 2182A nanovoltmeter set to the 10 mV input range. It featured an input impedance of >10 GΩ, hence, it was not loading the bridge. On the other hand, its own noise equaled a 1 kΩ resistor. To suppress the influence of line voltage, the sample rate was set to multiples of power line cycles (NPLC), with a maximum possible sample rate of 25 NPLC. Furthermore, all filters on the nanovoltmeter were turned off. To prevent the influence of temperature fluctuations, the measurements were performed in a temperature-controlled environment at 23 ± 1 °C and direct air drafts avoided.

The time series data for each measurement were captured over multiple hours and logged to a file. Since the sample rate slightly varied with the line frequency, the measurement was corrected for equally spaced samples using interpolation. The equidistant samples were then processed using the Welch algorithm, resulting in the power spectrum. The square root of the power spectrum then represented the noise in nV/√(Hz) format.

#### 4.2.4. Results

In the following section, the measured results are presented. First, the results from setup 1 are given, followed by the ones of setup 2. Afterwards, the results from both setups are combined to show the noise over a larger frequency range.

##### Results from Setup 1

**Figure 7:**
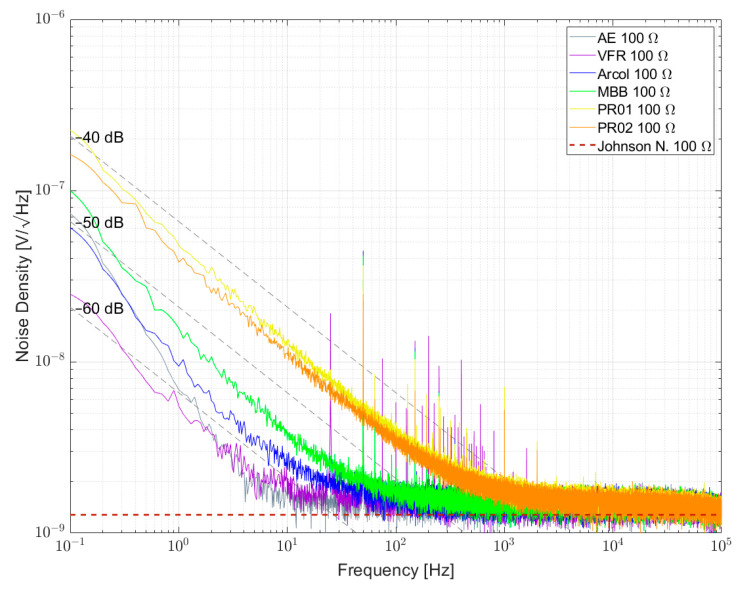
Noise spectral density for some 100 Ω resistors biased with 5 V per element.

**Figure 8:**
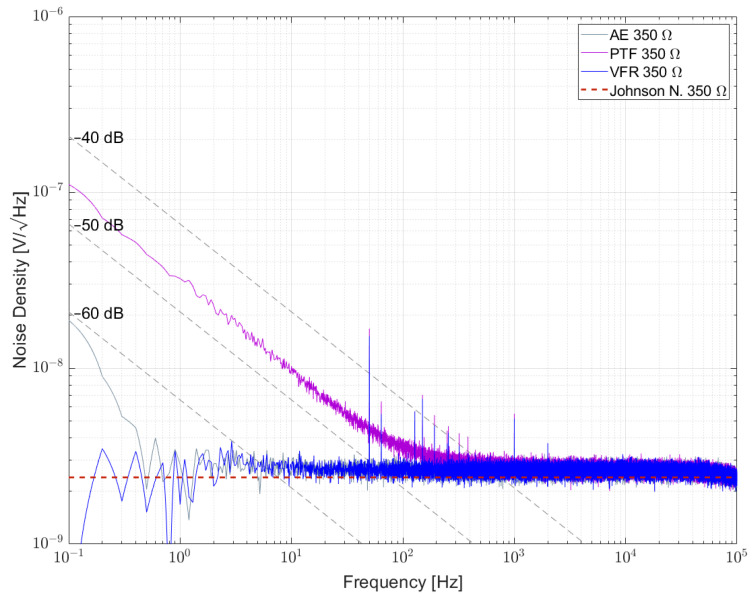
Noise density for some 350 Ω resistors biased with 5 V per element.

**Figure 9:**
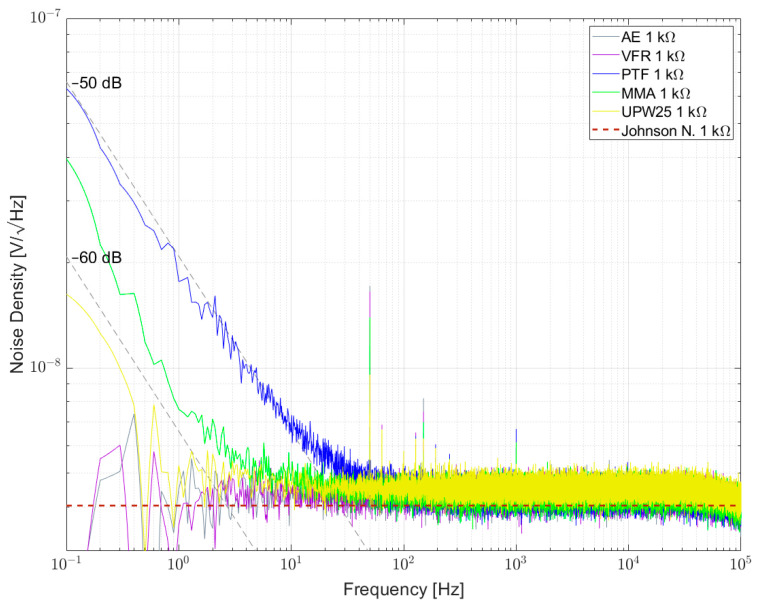
Noise spectral density for some 1 kΩ resistors biased with 5 V per element.

Figure 7, Figure 8 and Figure 9 show the measured results of excess noise with setup 1 on single resistors arranged in a bridge with resistance values of 100 Ω, 350 Ω and 1 kΩ, respectively. Table 6 recaps the calculated NI values for every resistor. For the 100 Ω resistors in Figure 7, AE 100R and VFR 100R show the smallest excess noise, with NI smaller than −60 dB. Most excess noise can be found in PR01 100R and PR02 100R, with NI of about −45 dB. It can be seen that, especially between 0.1 Hz and 1 Hz, the resistors are superimposed by 1/f^α^-noise with α > 1. Figure 8 shows that for AE 350R and VFR 350R, the setup from setup 1 reaches its limitation. The NI is better than −60 dB, but no certain number can be given. In Figure 7 AE 1k, VFR 1k and UPW25 1k reached the limitation of the setup too. The largest excess noise was measured for the PTF 1k resistor. Within one resistor series, all NI for different resistor values were comparable.

**Figure 10:**
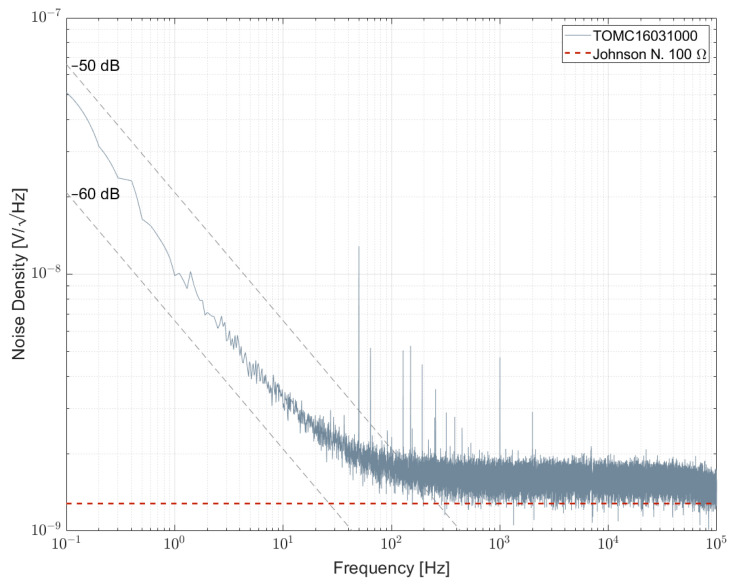
Noise spectral density for a 100 Ω resistor network biased with 5 V per element.

**Figure 11:**
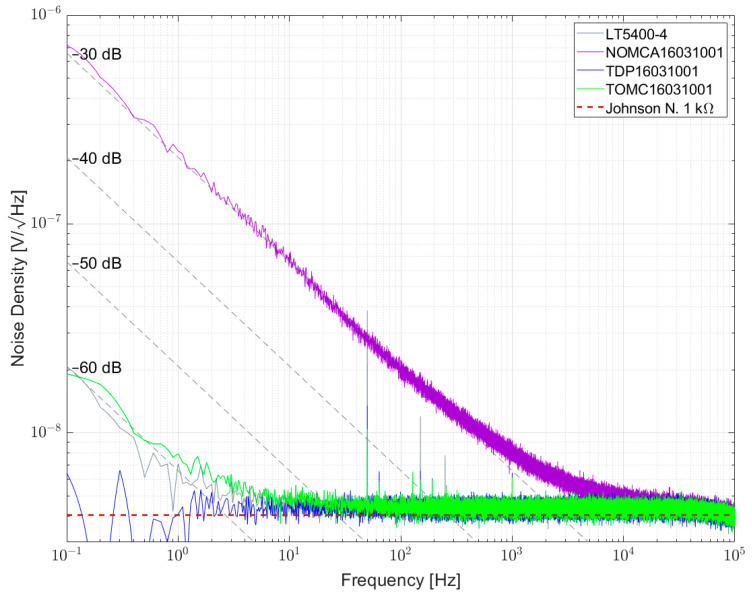
Noise spectral density for some 1 kΩ resistor networks biased with 5 V per element.

Figure 10 and Figure 11 show the measurement results of resistor networks analyzed with measurement setup 1 and their respective NI values given in Table 7. Figure 10 shows the TOMC16031000 resistor network with NI of about −59 dB. As for the 100 Ω single resistors, the spectrum is superimposed with 1/f^α^-noise with α > 1. Measurement results for 1 kΩ resistor networks are shown in Figure 11. TDP16031001 shows the least excess noise and cannot be determined quantitatively due to reaching the limit of the setup. NOMCA16031001 shows the largest excess noise with NI of about −30 dB, which equals the value given in the datasheet.

**Figure 12:**
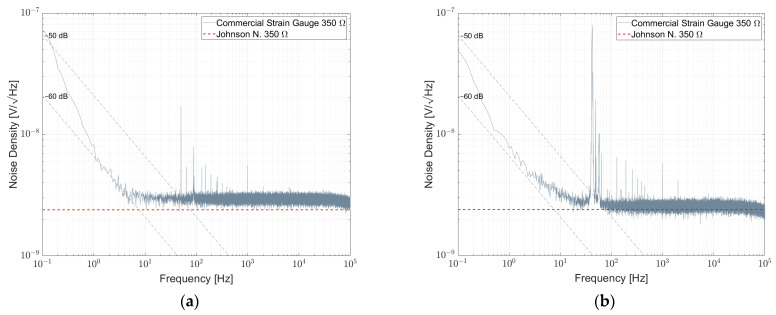
Noise spectral density for a commercial 350 Ω strain gauge (**a**) 1-LY11-6/350 free of strain and (**b**) 1-XY33-6/350 attached to an aluminum substrate.

Figure 12a shows the result of a commercial strain gauge 1-LY11-6/350. The strain gauges in the bridge were free of strain. Down to 1 Hz, the density spectrum follows 1/f. Between 0.1 Hz and 1 Hz, the spectrum is superimposed by 1/f^α^ -noise with α > 1. A second bridge with 1-XY33-6/350 strain gauges attached to an aluminum substrate was measured and shown in Figure 12b. They show a similar behavior in this frequency range. NI values for commercial strain gauges are shown in Table 8.

The printed strain gauge could not be measured with setup 1 since the output voltage of AD8429 went into its limits. It is probable that the single resistors of the bridge configuration differ too much, which created a large offset voltage at the input of the LNA and drove the output into saturation.

##### Results from Setup 2

**Figure 13:**
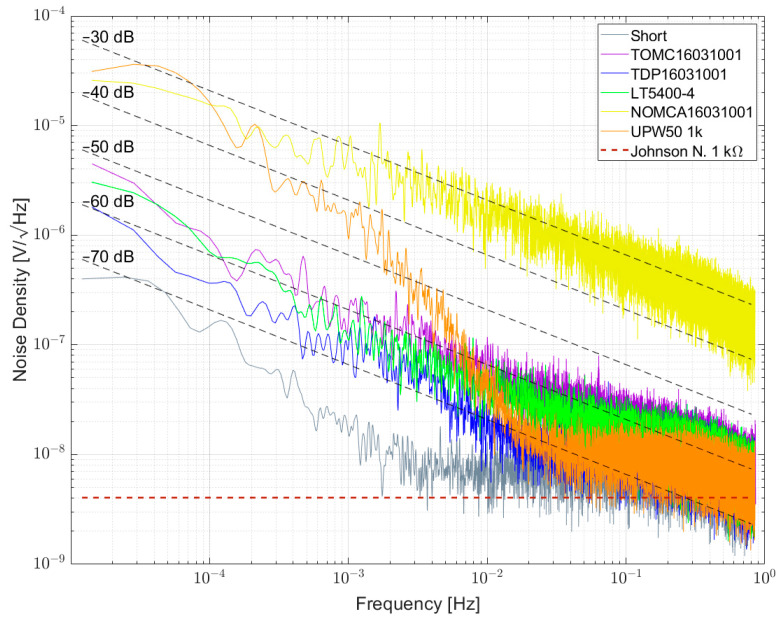
Noise spectral density for some 1 kΩ resistors and resistor networks biased with 5 V per element.

Figure 13 shows the noise spectral density for some 1 kΩ resistors and resistor networks. The lowest noise was found for the TDP16031001 network, directly followed by LT5400-4, both in the order of <−60 dB. As shown in previous papers, NOMCA16031001 exhibited the largest noise with −30 dB, matching the datasheet specification. A wirewound resistor type UPW50 was measured and excess noise was found.

**Figure 14:**
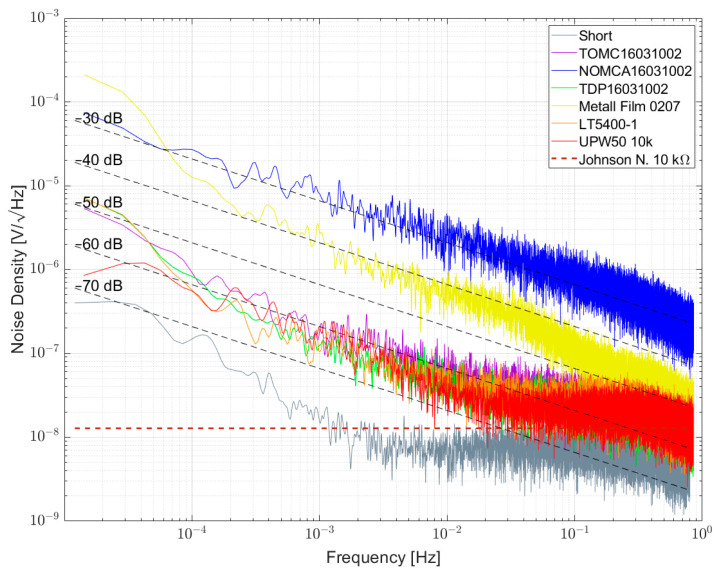
Noise spectral density for some 10 kΩ resistor networks biased with 5 V per element.

Figure 14 shows the noise spectral density plot for some 10 kΩ resistors and resistor networks. Here, TDP16031002 and LT5400-1 are on par with around −60 dB, which differs from the results given in [4], indicating variations between different samples and measurements. A wirewound resistor type UPW50 was measured and here the excess noise is on par with TDP16031002 and LT5400-1.

**Figure 15:**
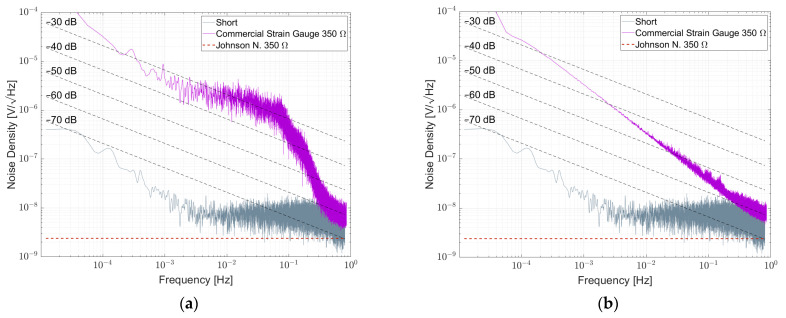
(**a**) Noise spectral density of a resistor bridge formed by four single-strain gauges biased with 5 V per element, (**b**) noise spectral density of a resistor bridge formed by four single-strain gauges that are attached to an aluminum substrate biased with 5 V per element.

In Figure 15a, the noise spectral density for a resistor bridge formed by four commercial single-strain gauges type HBK 1-LY11-6/350 intended for measurements on steel is shown. During the measurement, the resistive elements were not attached to a substrate and, thus, were free of strain. It is worth noting that besides 1/f-noise, the noise spectral density is superimposed by a 1/f^α^ component with α > 1. The noise index is in the order of −30 dB and thus comparable to the noise index of a NOMCA16031001 resistor network. On the other hand, the results of a similar strain gauge attached to a substrate with the proper adhesive are shown in Figure 15b. Here, the 1/f component almost vanished and the spectrum is dominated by 1/f^α^-noise with α > 1.

**Figure 16:**
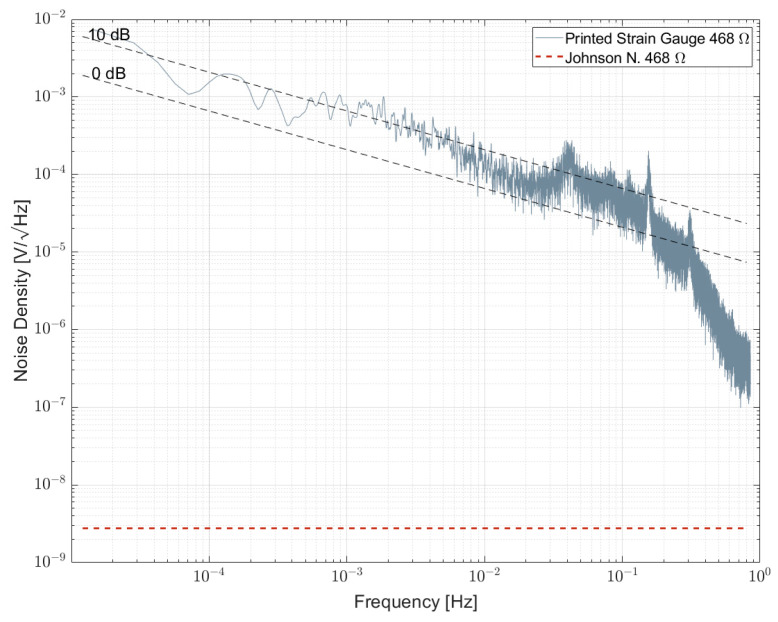
Noise spectral density of a resistor bridge formed by four single inkjet-printed strain gauges biased with 5 V per element.

A similar measurement was performed for the aforementioned inkjet-printed single-element strain gauge sensors. The noise spectral density is shown in Figure 16. Although limited by the upper frequency, a combination of 1/f-noise and 1/f^α^ noise with α>1 can be observed. The noise index is in the order of 10 dB and, thus, exceptionally large compared to other resistor technologies. This is probably due to the nature of resistive elements created by sintered silver nanoparticles.

**Figure 17:**
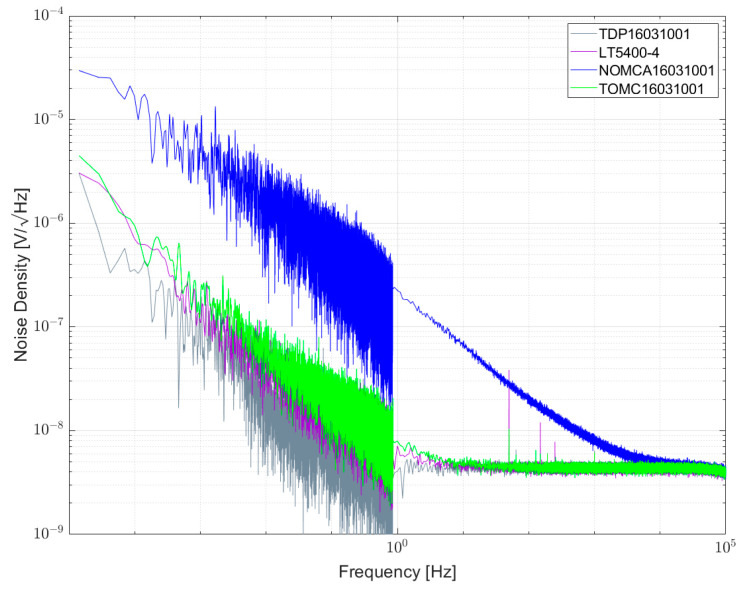
Combined noise spectral densities of some 1 kΩ resistor networks measured with setup 1 and 2.

In Figure 17, the measured results of 1 kΩ resistor networks of both setups are combined in one diagram. As can be seen, the noise spectral densities for NOMCA16031001, TDP16031001, TOMC16031001 and LT5400-4 are in good agreement, indicating that both setups lead to the same results.

## 5. Comparison with the Literature Results

**Figure 18:**
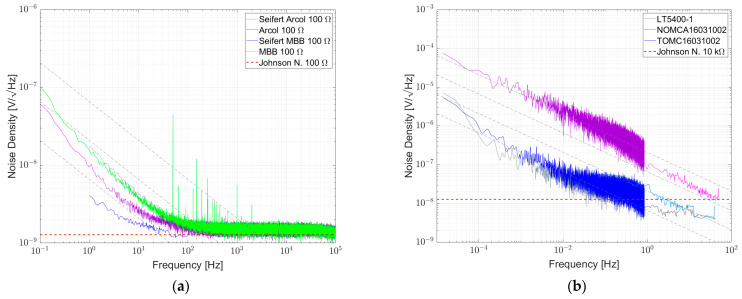
(**a**) Comparison of Seifert’s results for Arcol 100 Ω and MBB 100 Ω results with the results of the authors and (**b**) comparison of results from [41] for 10 kΩ resistor networks LT5400-1, NOMCA16031002 and TOMC16031002.

Comprehensive results of the individual parts can be found in [4,22,41]. Seifert gives sufficient information about the setup to estimate or read the NI of the single components and Beev [4] gives a range for NI values for individual components. Results from [22,41] were digitized and compared to our own. Arcol 100 Ω and MBB 100 Ω were used in Seifert’s paper. As shown in Figure 18a, the respective curves do not match our own results. Arcol 100 Ω was measured noisier in Seifert’s work while MBB 100 Ω was measured noisier here. For resistor networks, shown in Figure 18b, our own results on LT5400-1, NOMCA16031002 and TOMC16031002 are in fair agreement with [41], while good matching is given for LT5400-1. For NOMCA16031002, the results are not far apart; our measurement shows slightly more excess noise. For TOMC16031002, it is the opposite way and our results are slightly lower in noise. These small differences could probably be explained by variations from sample to sample.

## 6. Discussion

Different papers on noise measurements of resistors were reviewed and analyzed based on criteria such as bridge setting, supply voltage and others. This work gives an overview of existing methods as well as an insight in the theory behind such measurements. The standard method is limited in its ability to measure noise in modern resistors. On the other hand, improved setups have been proposed, resulting in the ability to measure much lower noise in resistors based on bridge configurations. Improvements were achieved via bipolar DC or AC bridge excitation and/or cross-correlation methods. The setup proposed by [4] is superior over the others. It has the largest flexibility with respect to the resistor values to be measured and uses cross-correlation for improved resolution. Temperature stabilization allows for very long measurements and the setup can also be used for very-low-frequency noise measurements. However, it is a custom setup, while most other setups use commercially available equipment. Nevertheless, it is hard to make any recommendations on which setup should be used for a certain type or technology of resistor, since for most setups the noise floor is unknown and only a few measurement results are available.

### 6.1. Reproduction of Measurement Results

In the present work, measurements on resistors and resistor networks were performed with two different setups to reproduce results from previous publications. A first setup, a variant of [22], used DC bridge excitation, a low-noise amplifier and the FFT functionality of a digital oscilloscope to measure the bridge voltage, while a second setup for measuring very low excess noise used a commercial nanovoltmeter. The advantage of setup 2 was the ability to measure bridges with higher imbalance, which led to larger offset voltages and saturated the LNA of setup 1. While setup 1 is good for components showing significant noise for frequencies up to 100 kHz, setup 2 is feasible for very-low-noise resistors, making it necessary to measure down to very low frequencies. However, it is worth noting that setup 1 can be used for very-low-frequency measurements too, but is limited by the 1/f noise of the LNA. A differential, very-low-noise chopper amplifier with JFET input stage could overcome the 1/f noise limit. Several different approaches for such chopper-based LNAs have already been proposed in the literature.

The results obtained from both setups on the same resistors show conclusive and matching results. The noise indices for commercial resistors and resistor networks calculated from these measurements agree with previous publications. This indicates that both setups are adequate to characterize excess noise in resistors. However, slight differences, probably due to sample variation, were observed. While setup 1 requires an LNA, setup 2 uses commercial test equipment only. However, with the NPLCs used in the measurements of setup 2, the maximum frequency is limited to 0.5 Hz. To overcome this limit, the NPLC could be increased up to 0.1 NPLC on a Keithley 2182A or by using another nanovoltmeter such as an Agilent 34420A with sampling rates up to 0.02 NPLC, with the disadvantage of a higher noise floor.

In general, there are only a few NI values in the literature given at all. This makes it hard to estimate how big the sample-to-sample variation is as well as by how much the setup or maybe other unknown parameters affect the results. The effect of sample variations for resistors in general and the reproducibility of noise measurements on the same component could be investigated.

### 6.2. Measurements on Commercial and Inkjet Printed Strain Gauges

Additionally, a few commercial strain gauges were measured to understand how they compare to different existing resistor technologies. Thin film strain gauges made of constantan (typically Cu55Ni44Mn1) on polyimide demonstrated a noise index similar to resistor networks made of tantalum nitride resistor films on high-purity alumina substrates, such as NOMCA1603. It is conspicuous that besides excess noise, the strain-free strain gauges demonstrated 1/f^α^ noise with α > 1. The reason for that should be investigated further to extract the nature of that extra noise contribution. This could be done by measuring the strain gauges prior and post-attaching them to a substrate.

A first inkjet-printed strain gauge made of sintered silver nanoparticles on a polyimide foil was measured and exhibited a 100 times larger noise index compared to the commercial strain gauge. Besides that, it also exhibited the very same 1/f^α^ noise with an α > 1 component, with the root cause for that being yet unknown, but obviously representative for strain gauges. Hence, a systematic investigation on inkjet-printed strain gauges should be performed to separate influencing parameters from printing and sintering and their impact on the noise spectral density, which is part of current work in progress.

### 6.3. Suggestion for Better Intercomparisons of Noise Spectral Density Plots

While the noise index is appropriate to directly compare results of different resistors, measured with different setups by a single number, the noise spectral density plots are not suitable unless the noise indices are plotted within the very same graph too, which is not always the case. This is due to the fact that the noise spectral density plot is dependent on the voltage the resistor is biased with. One way to overcome this issue is to make transparent how the noise indices are calculated and plotted within the graph, in this work presented by (12) and (17).

**Figure 19:**
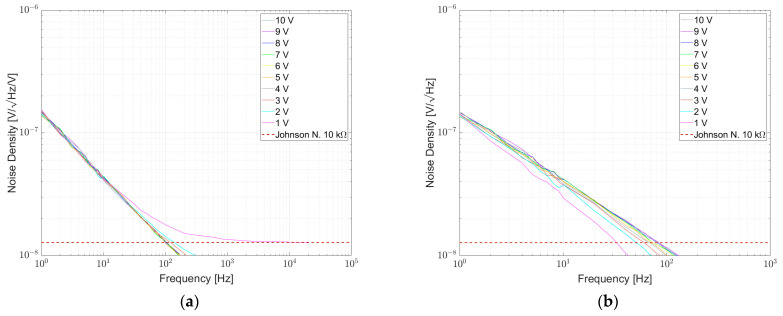
(**a**) Normalized noise spectral density of measurement results presented by [22] and (**b**) normalized noise spectral density of measurement results presented by [22], with the thermal noise removed.

Another way to solve this issue is to present the noise spectral density plots normalized to the bias-voltage of the resistor together with the thermal noise boarder. This only has the drawback that the thermal noise of the measurement is also normalized. However, since investigations are mainly addressing the 1/f component in resistors, this drawback is something that can be accepted. In Figure 19a, such a normalization was performed for results presented by [22]. It is obvious that such a normalized representation of a noise spectral density plot is independent from the voltage the resistor is biased with. Hence, it is easier to directly compare results of setups with different settings.

In case users do not feel comfortable with the obviously wrong representation of the thermal noise, its contribution to the normalized noise spectral density can be removed completely, as depicted in Figure 19b.

## Figures and Tables

**Table 2:** Some results from the papers of Seifert [22] and Beev [4].

NI [dB]	Resistors From [22]		Resistor Networks From [4]	
≥−40	MIRA Electronic 1206, 1%, 100 ppm, 100 Ω	Philips RC01, 5%, 200 ppm, 100 Ω	NOMCA	
	Vitrohm RGU 526-0, 2%, 100 ppm, 100 Ω	Phoenix PR01, 5%, 250 ppm, 100 Ω	AORN	
	Mira 0805, 1%, 100 ppm, 100 Ω	Philips RC01, 5%, 200 ppm, 10 kΩ	T914	
	Panasonic ERJ-8ENF, 1%, 100 ppm, 10 kΩ	Mira 0805, 1%, 100 ppm, 10 kΩ	ACAS	
−40 to −60	Microtech CMF0805, 0.1%, 25 ppm, 100 Ω	Vishay SMM0204-MS1, 1%, 50 ppm, 100 Ω	PRA	
	Yageo MF0207, 1%, 100 ppm, 1 kΩ	Arcol MRA 0207, 0.1%, 15 ppm, 100 Ω	DFN	
	Vishay Dale CMF55, 0.1%, 25 ppm, 1 kΩ	Vishay MMA0204, 0.1%, 15 ppm, 10 kΩ	DIP-1999	
	Vitrohm ZC0204, 1%, 50 ppm, 10 kΩ		VSOR	
	Tyco RN73, 0.1%, 10 ppm, 10 kΩ		MAX549x	
≤−60	Vishay Beyschlag MMA0204, 1%, 50 ppm, 100 Ω	Phycomp TFx13 series, 0.1%, 25 ppm, 100 Ω	NOMC	PRND
	Ohmite, 5%, 100 Ω	Vishay Beyschlag MBB0207, 1%, 50 ppm, 100 Ω	MORN	RIA
	Welwyn RC55Y, 0.1%, 15 ppm, 1 kΩ	Yageo PO 593-0, 5%, 200 ppm, 100 Ω	OSOP	ORN
	Vishay Beyschlag MMA0204, 1%, 50 ppm, 100 Ω	Phycomp TFx13 series, 0.1%, 25 ppm, 100 Ω	TDP	HTRN
	Ohmite, 5%, 100 Ω	Vishay Beyschlag MBB0207, 1%, 50 ppm, 100 Ω	DIV23	MPM
	Welwyn RC55Y, 0.1%, 15 ppm, 1 kΩ	Yageo PO 593-0, 5%, 200 ppm, 100 Ω	SMN/SMNZ	LT5400

**Table 3:** The analyzed single resistors and their parameters from datasheets.

Manufacturer Part Number	Abbreviation	Value [Ω]	Tolerance [%]	Power [W]	Technology
AlphaElectronics MC Y 000100 T	AE 100R	100	±0.01	0.3 (at 125 °C)	-
AlphaElectronics MC Y 1K0000 T	AE 1k	1000	±0.01	0.3 (at 125 °C)	-
AlphaElectronics MC Y 000350 T	AE 350R	350	±0.01	0.3 (at 125 °C)	-
Arcol MRA 0207	Arcol 1k	100	±0.1	0.25	Metal film
Vishay Beyschlag MBB 0207	MBB 100R	100	±1	0.6 (at 70 °C)	Thin film
Vishay Beyschlag MMA 0204	MMA 1k	1000	±1	0.4 (at 70 °C)	Thin film
Vishay Bccomponents PR01	PR01 100R	100	±5	1 (at 70 °C)	Metal film
Vishay Bccomponents PR02	PR02 100R	100	±5	2 (at 70 °C)	Metal film
Vishay Dale PTF-56-1K0000	PTF 1k	1000	±0.1	0.125 (at 85 °C)	Metal film
Vishay Dale PTF-56-350R00	PTF 350R	350	±0.1	0.125 (at 85 °C)	Metal film
Neohm UPW25	UPW25 1k	1000	±0.1	0.25 (at 125 °C)	Wirewound
Vishay Foil Resistors S Series (S102J)	VFR 100R	100	±0.01	0.6 (at 70 °C)	Bulk Metal^®^ Foil
Vishay Foil Resistors S Series (S102J)	VFR 1k	1000	±0.01	0.6 (at 70 °C)	Bulk Metal^®^ Foil
Vishay Foil Resistors S Series (S102J)	VFR 350R	350	±0.01	0.6 (at 70 °C)	Bulk Metal^®^ Foil
YAGEO 10.0K 0207	MetalFilm	10,000	±1	0.6 (at 70 °C)	Metal film

**Table 4:** Analyzed resistor networks and their parameters from datasheets.

Manufacturer Part Number	Abbreviation	Value [Ω]	Tolerance Absolute [%]	Power/Resistor [W]	Technology	Material
LT5400-1	LT5400-1	10,000	±15	0.8	-	Chromium silicide on silicone substrate [4]
LT5400-4	LT5400-4	1000	±15	0.8	-	Chromium silicide on silicone substrate [4]
Vishay Dale NOMCA-1603-1002	NOMCA16031002	10,000	±1	0.1 (at 70 °C)	Thin film	Tantalum nitride on alumina substrate
Vishay Dale NOMCA-1603-1001	NOMCA16031001	1000	±1	0.1 (at 70 °C)	Thin film	Tantalum nitride on alumina substrate
Vishay Thin Film TDP-1603-1002	TDP16031002	10,000	±0.1	0.8 (at 70 °C)	Thin film	Passivated nichrome on silicone/alumina substrate
Vishay Thin Film TDP-1603-1001	TDP16031001	1000	±0.1	0.8 (at 70 °C)	Thin film	Passivated nichrome on silicone/alumina substrate
Vishay Dale TOMC-1603-1002	TOMC16031002	10,000	±1	0.1 (at 70 °C)	Thin film	Passivated nichrome
Vishay Dale TOMC-1603-1001	TOMC16031001	1000	±1	0.1 (at 70 °C)	Thin film	Passivated nichrome
Vishay Dale TOMC-1603-1000	TOMC16031000	100	±1	0.1 (at 70 °C)	Thin film	Passivated nichrome

**Table 5:** Analyzed strain gauges and their parameters.

Manufacturer Part Number	Abbreviation	Value [Ω]	Tolerance [%]	Technology	Material
HBK 1-LY11-6/350	Comm. strain gauge	350	±0.35	-	Constantan on polyimide
HBK 1-XY33-6/350	Comm. strain gauge	350	±0.35	-	Constantan on polyimide
Printed Strain Gauge	Printed strain gauge	468	-	Inkjet printing	Silver ink on polyimide

**Table 6:** Noise indices for 100 Ω, 350 Ω and 1 kΩ resistors measured with setup 1.

Resistor	Noise Index [dB]
AE 100R	−64
VFR 100R	−63
Arcol 100R	−59
MBB 100R	−55
PR01 100R	−45
PR02 100R	−46
AE 350R	<−60
PTF 350R	−47
VFR 350R	<−60
AE 1k	<−60
VFR 1k	<−60
PTF 1k	−51
MMA 1k	−57
UPW25 1k	<−60

**Table 7:** Noise indices for 100 Ω and 1 kΩ resistor networks measured with setup 1.

Resistor Network	Noise Index [dB]
TOMC16031000	−59
LT5400-4	−53
NOMCA16031001	−31
TDP16031001	<−60
TOMC16031001	−57

**Table 8:** Noise index for 350 Ω commercial strain gauge.

Strain Gauge	Noise Index [dB]
Comm. strain gauge 1-LY11-6/350	−60
Comm. strain gauge 1-XY33-6/350	−61

## Data Availability

Measurement data from [4,22,41] were used for comparison to our own measurements.
